# Molecular analysis of RAX2-regulated retinal development using human retinal organoids at a single-cell resolution

**DOI:** 10.3389/fcell.2025.1609826

**Published:** 2025-06-05

**Authors:** Shaojun Wang, Yi Sun, Jie Na, Yue Huang, Guang Liu

**Affiliations:** ^1^ Senior Department of Ophthalmology, 3rd Medical Center of Chinese PLA General Hospital, Beijing, China; ^2^ State Key Laboratory of Common Mechanism Research for Major Disease, Institute of Basic Medical Sciences, Chinese Academy of Medical Sciences and Peking Union Medical College, Beijing, China; ^3^ Department of Medical Genetics, Institute of Basic Medical Sciences, Chinese Academy of Medical Sciences and Peking Union Medical College, Beijing, China; ^4^ Center for Regeneration, Aging and Chronic Diseases, School of Basic Medical Sciences, State Key Laboratory for Complex, Severe and Rare Diseases, Tsinghua University, Beijing, China

**Keywords:** human embryonic stem cells (hESC), retinal organoid, retinal development, photoreceptor cells, ScRNA-seq

## Abstract

Human embryonic stem cells (hESC)-derived retinal organoids are sophisticated *in vitro* systems for dissecting the complex dynamics of human retinal development. The formation of the human retina is a precisely organized process that depends on the regulated differentiation of retinal progenitor cells; however, many of the basic mechanisms remain to be explored. Here, using hESC-derived retinal organoids, we elucidated the temporal contribution of RAX2 to retinal development, with an emphasis on photoreceptor cells (PC) formation. The results were corroborated using human fetal retinal tissue at various gestational ages. Using CRISPR/Cas9-mediated gene knockout, we delineated the essential role of RAX2 in modulating PC specifications. *RAX2* deficiency significantly altered the expression of *PAX6* and *SOX2*, two essential regulators of retinogenesis. Our results suggested that RAX2 is significant in retinal development, underpinning its potential as a therapeutic target in related retinal disorders.

## 1 Introduction

The human retina, acting as a processor for integrating visual signals, orchestrates interactions among various retinal cell types in a delicate cellular structure. Retinogenesis is the process by which multipotent retinal progenitor cells (RPC) differentiate into specialized cells, including retinal ganglion cells (RGC), photoreceptor cells (PC, including rods and cones), Müller cells (MC), amacrine cells and bipolar cells. This process is meticulously orchestrated by a network of signaling pathways, as delineated in previous studies (Bassett and Wallace, 2012). Recent studies using bulk transcriptomic profiling, single-cell RNA sequencing (scRNA-seq), and single-cell assay for transposase-accessible chromatin sequencing (scATAC-seq) have systematically examined the cellular composition and molecular expression patterns of the human retina and retinal organoids (RO) derived from human embryonic stem cells (hESC) (Voigt et al., 2021; Li et al., 2023; Wahle et al., 2023; Zhang et al., 2024). These investigations have provided critical insights into the spatiotemporal dynamics of cellular diversification, thereby offering an integrative framework for understanding the molecular mechanisms underlying retinogenesis and retinal disease pathogenesis. In a previous study, using scRNA-seq to analyze hESC derived RO at five different time points (day36-day186, D36-D186), we identified 9 cell populations, including RPC, RGC, PC, MCs, and retinal pigment epithelial (RPE) cell populations, and described the emergence, maturation, and regulation of RPC and PC populations in detail (Wang et al., 2021).

The retinal and anterior neural fold homeobox (RAX) gene family encodes homeodomain transcription factors, and is crucial for vertebrate retinal development. Through evolutionary analysis, jawed vertebrate *RAX* genes were classified into two distinct subgroups: *RAX1* (commonly referred to as *RAX*) and *RAX2* (Kon and Furukawa, 2020). RAX is initially expressed in the anterior neural fold and later in the embryonic diencephalon, which gives rise to the retina and pineal gland (Mathers et al., 1997). RAX is critical for retinal cell fate determination and the maturation and survival of PC (Irie et al., 2015). *RAX*-deficient mice exhibit severe forebrain malformations and lack optic vesicles (Mathers et al., 1997). Mutations in human *RAX* have been linked to congenital ocular disorders, including anophthalmia and microphthalmia (Voronina et al., 2004). RAX2 (also known as QRX) is required for retinal neurogenesis in *Xenopus* (Wu et al., 2009) and chicks (Sanchez-Arrones et al., 2009). Studies have found that *RAX2* orthologs are essential for maintaining adult medaka fish retinal stem cells (Reinhardt et al., 2015). RAX2 protein physically interacts with the CRX protein synergistically to modulate the expression of PC-specific genes, such as *Rhodopsin*. Emerging clinical evidence has linked RAX2 mutations to various inherited retinal diseases (IRD). Dominant mutations, such as c.260G>A (p.Arg87Gln), have been associated with age-related macular degeneration (AMD), while variants like c.409G>C (p.Gly137Arg) and c.417_422dup (p.Pro140_Gly141dup) have been linked to cone-rod dystrophy (CRD) (Wang et al., 2004). The heterozygous c.465_475del (p.Ala156Argfs*131) variant, identified in familial cases of cone dystrophy or CRD, disrupts the N-terminal coding region of RAX2, potentially impairing its function as a CRX cofactor (Yang et al., 2015). Van de Sompele *et al.* demonstrated that biallelic *RAX2* mutations, including c.155C>G (p.Pro52Arg), c.335dup (p.Ala113Glyfs*178), c.145 T>C (p.Ser49Pro), and g.3771337_3774298del, cause autosomal recessive retinitis pigmentosa (ARRP) (Van de Sompele et al., 2019). These mutations may impair the RAX2 protein folding, stability, and transactivation capability. Notably, RAX2 mutations are not compensated by RAX activity in human disease. Unlike humans, mice lack *RAX2* orthologue, complicating functional studies (Wang et al., 2004). ScRNA-seq analysis of the human fetal neural retina revealed that RAX2 was primarily expressed in PC (Hu et al., 2019), which aroused our interest in exploring its potential role in human retinogenesis.

## 2 Materials and methods

### 2.1 Patients and tissue samples

The five human retinal specimens used in this study were obtained from voluntarily donated aborted fetuses, sourced from the Senior Department of Ophthalmology at the Third Medical Center of the Chinese PLA General Hospital. The Ethics Committee of the Third Medical Center of the Chinese PLA General Hospital approved this study (ID: KY 2021-021), and written informed consent was obtained from all participants. The procedures in this study adhered to the Helsinki Declaration of 1964 and its amendments, ensuring ethical integrity (World Medical, 2013).

### 2.2 hESC culture and RO differentiation

The hESC line H9 were routinely cultured in Essential 8 medium (ThermoFisher, A1517001) on plates coated with Vitronectin (Gibco, A14700). For passaging, cells were treated with Accutase (Stemcell Tech, 07920). RO differentiation followed established protocols with minor modifications (Wang et al., 2021; Kuwahara et al., 2015). Aggregates were cultured under 40% O_2_/5% CO_2_ conditions (30 aggregates per 10-cm dish) from day 24 (D24), using an NR-differentiation medium comprising DMEM/F12 (Gibco, 10565018), KSR (Gibco, 10828028), N2 supplement (Gibco, A1370701), 0.1 mM taurine (Sigma, T0625), and 0.5 μM retinoic acid (Sigma, R2625). Under these conditions, RO continued to grow for several weeks.

### 2.3 Establishment of genetically engineered hESC

Single guide RNAs (sgRNA) constructs targeting critical *RAX2* were cloned into px459 plasmids (Addgene, 62988) for knockout cell generation. HESC were transfected with these sgRNA plasmids using the Lipofectamine Stem Transfection Reagent (Invitrogen, STEM00001) and exposed to 0.5 μg/mL puromycin for 48 h 2,000–3,000 surviving cells were plated on a 6 cm dish, and 96 single colonies were picked up to a 96-well plate. Genomic DNA was extracted for PCR using specific primers:

Fw: CTTAGGGCGTGAGAAGGGAT;

Rv: CCCCACGCCCAATTAACAGA.

The PCR products were validated by TA cloning and Sanger sequencing to confirm *RAX2* gene deletions.

## 3 Results

### 3.1 Highly-expressed RAX2 in PC within RO and human fetal retinal tissue

**FIGURE 1:**
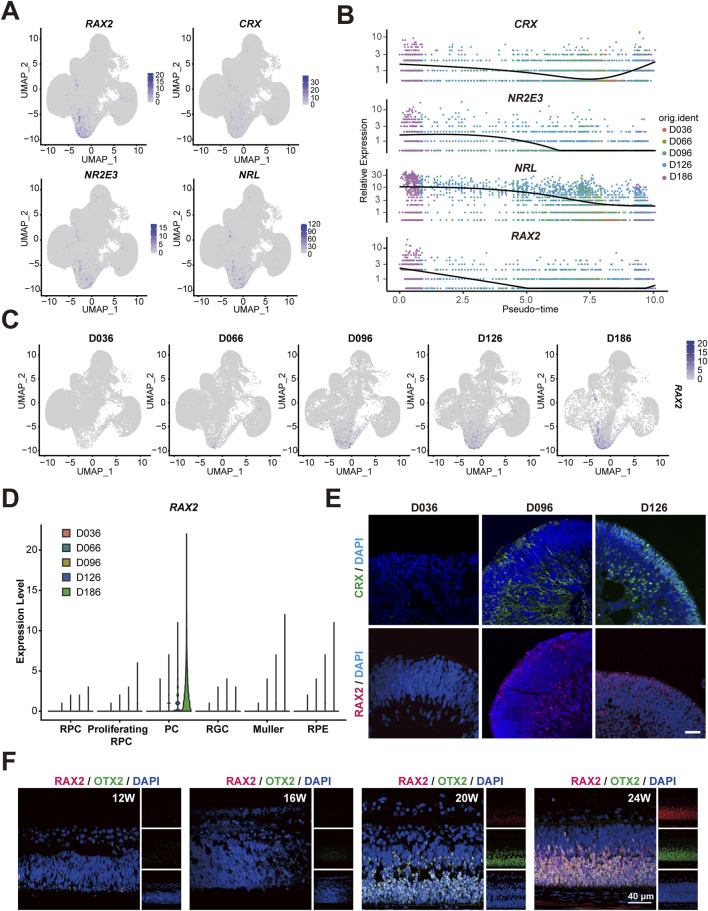
Highly expressed *RAX2* in PC during early stages of RO and tissue. **(A)** Multiple feature plots of RO display integrated expression profiles across five timepoints (D036, D066, D096, D126, D186), highlighting the expression of *RAX2* and the hallmark genes of the PC population, including *CRX*, *NRL* and *NR2E3*, in hESC-derived RO. **(B)** Pseudotemporal trajectory map exhibiting the expression of *RAX2* and marker genes of PC population, including *CRX*, *NRL*, and *NR2E3* in hESC-derived RO at different timepoints (D036, D066, D096, D126, D186). **(C)** Multiple feature plots exhibiting *RAX2* expression in hESC-derived RO at different timepoints (D036, D066, D096, D126, D186). **(D)** Violin plots exhibiting *RAX2* gene expression in hESC-derived RO at different timepoints (D036, D066, D096, D126, D186). **(E)** Representative IF-staining images of CRX and RAX2 in hESC-derived RO at different timepoints (D036, D096, D126). Scale bars, 40 μm. **(F)** Representative IF-staining images of RAX2 and OTX2 in human retinal tissue from aborted fetuses, spanning gestational ages of 12–24 weeks. Scale bars, 40 μm. W: weeks.

Our earlier investigation used an *in vitro* self-organization model of human RO derived from hESC, which mimicked human retinal development, to conduct an scRNA-seq analysis at five different time points during RO differentiation (D36, D66, D96, D126, and D186) (Wang et al., 2021). In this study, to delineate the role of the RAX2 in retinogenesis, we reanalyzed the scRNA-seq data. Canonical markers were used to distinguish 6 cell clusters: RPC, Proliferating-RPC, PC, RGC, MCs and RPE cells (Supplementary Figures S1A, S1B). RAX2 was primarily detected in the PC population (Figure 1A). A gradual increase in *RAX2* expression correlating with PC emergence in RO was observed (Figures 1B–D), consistent with the immunofluorescence (IF) staining of human RO, which also revealed a progressive increase in RAX2-positive cells (Figure 1E; Supplementary Figure S2A). Moreover, *RAX2* expression patterns aligned with canonical PC markers, including *CRX*, *NR2E3* and *NRL* (Figures 1A,B). CRX-positive cells appeared at D36 in a human RO culture and gradually increased over time. The expression of *NRL* and *NR2E3* significantly increased during the maturation of PC (Wang et al., 2021), and *OTX2* was found to be involved in embryonic PC fate determination (Muranishi et al., 2011). To enhance our comprehension of RAX2 dynamics in retinal development, we obtained human retinal tissue from voluntarily donated aborted fetuses aged 12–24 weeks of gestation, and performed multi-immunofluorescence (multi-IF) staining to precisely track the temporal expression patterns of RAX2 (Figure 1F; Supplementary Figure S2B). A notable increase in RAX2-positive cells was observed from 20 to 24 weeks, coinciding with the reported initiation of PC development (Hu et al., 2019). These findings indicated that RAX2 may regulate PC maturation during retinal development.

### 3.2 Establishment of *RAX2*-knockout hESC utilizing CRISPR/Cas9-mediated gene editing

**FIGURE 2:**
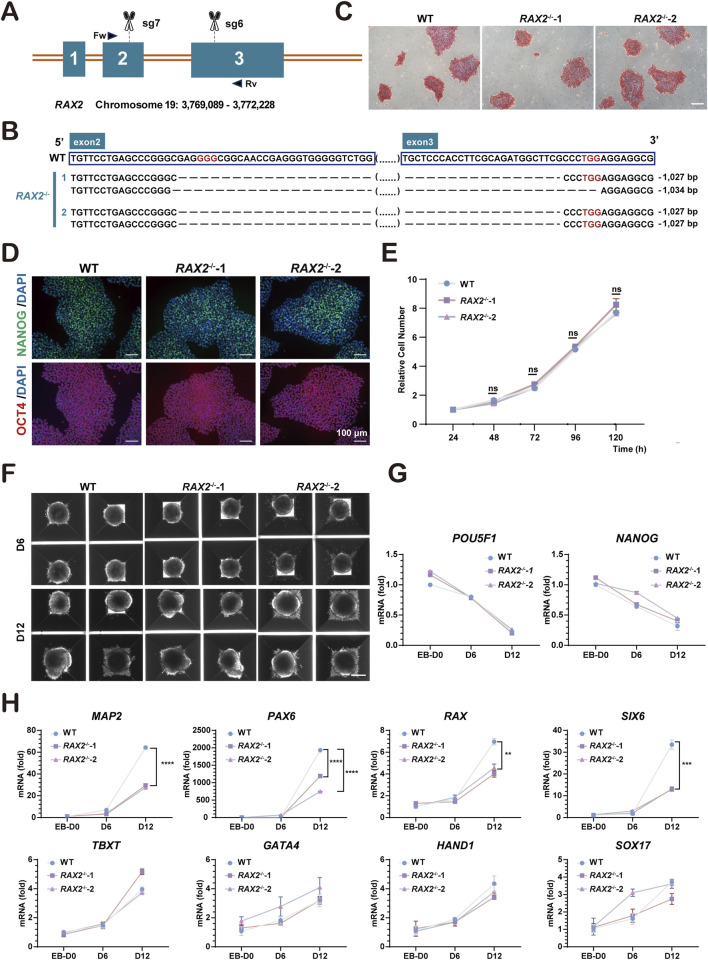
Establishment of *RAX2*-knockout hESC. **(A)** Schematic illustration of knocking out *RAX2* in hESC by CRISPR/Cas9 system. Scissors indicate the sgRNAs; boxes represent the exons; triangular arrows represent primers. Fw: forward primer; Rv: reverse primer. **(B)** Sanger sequencing results for *RAX2* ^−/−^ and WT hESC clones. Red words indicate the PAM sequence; ellipses in parentheses indicate sequences that are not listed; dashed line indicates deleted bases. **(C)** Alkaline phosphatase staining of *RAX2* ^−/−^ and WT hESC clones. Scale bars, 100 μm. **(D)** Representative IF-staining images of pluripotency markers in *RAX2* ^−/−^ and WT hESC clones. Scale bars, 100 μm. **(E)** Cell proliferation rate of *RAX2* ^−/−^ and WT hESC clones (n = 3 independent experiments). ns, not significantly different. **(F)** Representative images of EB formation assay for *RAX2* ^−/−^ and WT hESC clones. Scale bar, 200 μm. **(G)** RT-qPCR analysis for *POU5F1* and *NANOG* expression in *RAX2* ^−/−^ and WT hESC -derived EBs at different timepoints (D0, D6, D12). **(H)** RT-qPCR analysis for ectoderm, endoderm, and mesoderm markers expression in *RAX2* ^−/−^ and WT hESC -derived EBs at different timepoints (D0, D6, D12).

To explore the influence of RAX2 on human retinal development, the CRISPR/Cas9 system was used to disrupt critical exons of *RAX2* in hESC (H9 cell line). Seven sgRNAs were created to target different regions around the gene, and their effectiveness was evaluated using a surveyor assay (Supplementary Figure S3). Cas9/sgRNA-7 and Cas9/sgRNA-6, both of which exhibited notable cleavage efficiencies, were selected for subsequent gene editing (Figure 2A). Two homozygous mutants, *RAX2* ^−/−^-1 and *RAX2* ^−/−^-2, were successfully generated and validated through Sanger sequencing (Figure 2B). Evaluation of genomic copy number variation (CNV) (Supplementary Figure S4), ESC colony morphology (Figure 2C), pluripotency markers expression (Figure 2D), and cell proliferation (Figure 2E) showed no significant differences between *RAX2* ^−/−^ and wild type (WT) hESC. Embryoid body (EB) formation assay (Figures 2F,G) revealed that RAX2 deficiency in hESC significantly reduced the expression of ectoderm markers in the derived EBs, including *MAP2*, *PAX6*, *RAX*, and *SIX6* (Figure 2H). This finding underscored the essential function of *RAX2* in the ectoderm-related differentiation process.

### 3.3 *RAX2* deficiency affects PC fate determination during RO differentiation

**FIGURE 3:**
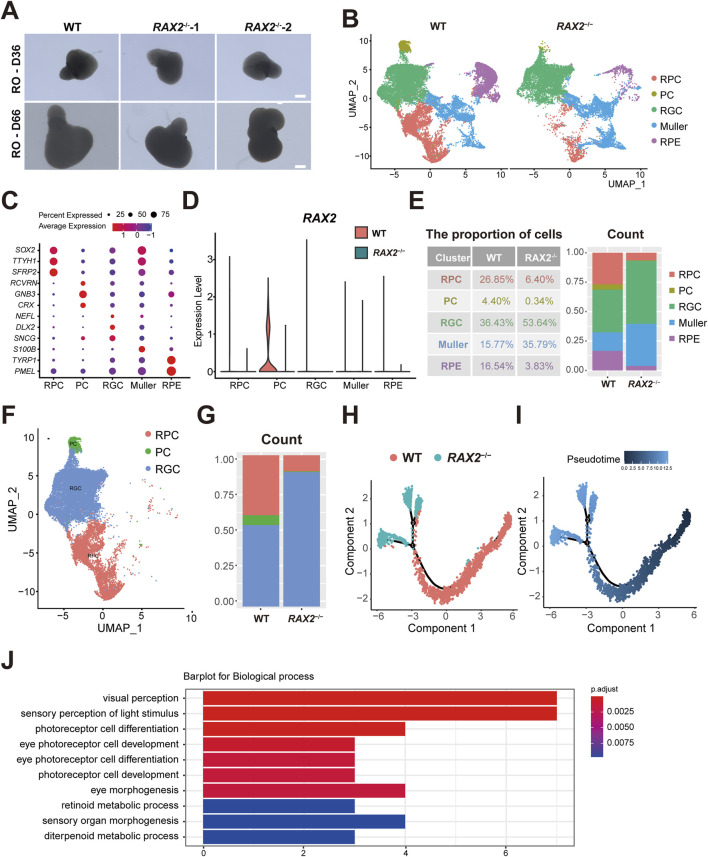
The absence of *RAX2* affects PC fate determination. **(A)** Representative images of *RAX2* ^−/−^ and WT hESC-derived human RO at D36 and D66. Scale bars, 100 μm. **(B)** UMAP plots of the *RAX2* ^−/−^ and WT hESC-derived RO in D66, labeled by cell types. **(C)** Dot plots for the marker genes expression by cell types. The color represents the average expression level; the size of dot represents the percentage of cells within a cell type. **(D)** Violin plots for *RAX2* expression in clusters from *RAX2* ^−/−^ and WT hESC-derived RO. **(E)** Proportion of each cell types from *RAX2* ^−/−^ and WT hESC-derived RO in D66. **(F)** UMAP plot of the RPC, PC and RGC clusters from the *RAX2* ^−/−^ and WT hESC-derived RO in D66, labeled by cell types. **(G)** Percentages of the RPC, PC and RGC clusters from the *RAX2* ^−/−^ and WT hESC-derived RO in D66. **(H)** The Monocle 2 trajectory plot showing the pseudotemporal ordering of cluster RPC, RGC and PC from *RAX2* ^−/−^ and WT hESC-derived RO in D66. Numbers in black circles indicate the different cell status numbers. **(I)** Pseudotemporal ordering trajectory map of RPC, RGC and PC clusters from *RAX2* ^−/−^ and WT hESC-derived RO in D66. The colors from dark to light indicate the pseudotime order. **(J)** GO analysis of the top 10 downregulated biological processes in PC subset from the *RAX2* ^−/−^ and WT hESC-derived RO at D66. Horizontal axis values the count of enriched genes per term.

Using a previously established BMP4-induced RO self-organization protocol (Wang et al., 2021), WT and *RAX2* ^−/−^ hESC were grown in a 3D culture for 66 days (Figure 3A), and twenty-four RO were harvested for scRNA-seq analysis from each of WT and RAX2^−/−^ group. Following a rigorous quality control evaluation and removal of doublets, a UMAP analysis revealed five primary cell clusters, with the cell types identified through enriched gene profiles and canonical markers (Figures 3B,C). A marked reduction in *RAX2* expression was detected in all the *RAX2* ^−/−^ hESC-derived RO cell clusters identified (Figure 3D). A significant decrease in the percentage of PC, RPC, and RPE cell populations was observed in RO derived from *RAX2* ^−/−^ hESC compared to the those from WT hESC (Figure 3E). Considering the process of retinal development, we focused on RPC, RGC, and PC clusters (Figures 3F,G). A developmental pseudotime trajectory analysis was conducted, which helped reveal highly interconnected nodes potentially indicating the differentiation status (Figures 3H,I). Depletion of *RAX2* significantly altered various cellular distributions. Differentiation into PC was notably affected by the absence of *RAX2*, leading to a bias towards RGC lineage commitment. Additionally, the PC population analysis revealed a decrease in pathways associated with PC differentiation (Figure 3J). RT-qPCR analysis demonstrated decreased expression of PC-specific markers (*CRX*, *NRL*, and *NR2E3*) in RO derived from *RAX2* ^−/−^ hESC, alongside elevated levels of RGC markers (*POU4F2* and *THY1*), consistent with the observed lineage bias (Supplementary Figure S5). These findings underscored the critical function of RAX2 in PC fate determination.

### 3.4 RAX2 regulates the expression of *PAX6* and *SOX2* during RO differentiation

**FIGURE 4:**
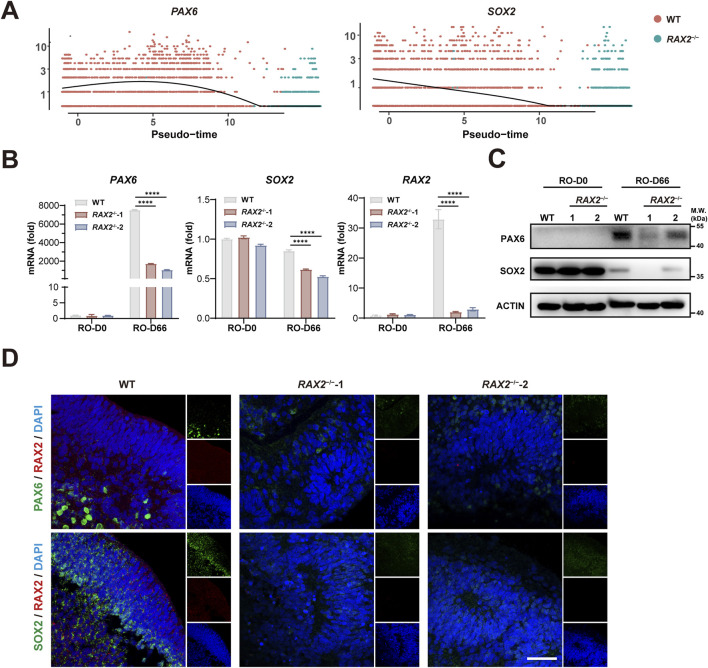
RAX2 regulates *PAX6* and *SOX2* expression. **(A)** Expression of *SOX2* and *PAX6* from *RAX2* ^−/−^ and WT hESC-derived RO over pseudotime. **(B)** RT-qPCR analysis for the expression of *RAX2*, *PAX6*, and *SOX2* in RO derived from *RAX2* ^−/−^ and WT hESC at D66. All experiments were repeated in three batches of organoids. **(C)** Expression of PAX6, and SOX2 in RO derived from *RAX2* ^−/−^ and WT hESC at D66 was detected by Western blot. All experiments were repeated in three batches of organoids. **(D)** Representative images of IF staining of RAX2, PAX6, and SOX2 in RO derived from *RAX2* ^−/−^ and WT hESC at D66. Scale bars, 40 μm. All experiments were repeated in three batches of organoids.

In our previous study, we observed that the proportion of each cell type, including PC, in *RAX2* ^−/−^ hESC-derived RO differed from that of the WT RO (Figure 3E), suggesting that RAX2 influenced the differentiation state of the entire organoid. To elucidate the underlying mechanism, we analyzed differentially expressed genes and noticed that the expression patterns of *PAX6* (Oron-Karni et al., 2008) and *SOX2* (Diacou et al., 2022), both vital for eye development, were significantly altered by *RAX2* deficiency (Figure 4A). RT-qPCR and Western blot analyses confirmed the reduced expression of PAX6 and SOX2 (Figures 4B,C). In addition, IF staining analysis of human RO at D66 revealed a marked decrease in the fluorescence intensity of PAX6 and SOX2 in *RAX2* ^−/−^ hESC-derived RO (Figure 4D). Overall, our results suggested that RAX2 is critical for retinal development by modulating *PAX6* and *SOX2* expression.

## 4 Discussion

In this work, we systematically examined the expression patterns of *RAX2* in human fetal retinal tissue and hESC-derived RO at different stages. By integrating bioinformatics analyses with biochemical assays of RNA and protein levels in *RAX2*-deficient hESC-derived RO, we delineated RAX2 as a pivotal determinant of PC specification. Notably, the loss of RAX2 significantly altered the proportions of various cell populations within the RO. The scRNA-seq results, validated through RT-qPCR, Western blotting, and IF staining, demonstrated that these alterations correlated with reduced expression of PAX6 and SOX2, which are key regulators in retinal development. The precise modulation of *PAX6* and *SOX2* expression within optic cup progenitors is essential for retina development, with a release of neural potential in the retina (Klimova and Kozmik, 2014; Oron-Karni et al., 2008). The spatial and temporal regulation of PAX6 expression, however, remains incompletely understood, suggesting that the regulatory function of RAX2 may be more complex than previously appreciated (Wang et al., 2004).

Our observations suggest that alterations in RAX2 expression are vital for retinal development, particularly in PC. Previous researches have shown the specific co-expression patterns of Rax2 and Vsx2 in defining retinal cell identity (Pandit et al., 2015), with external signals like BMP activity influencing RAX2 expression in chicks and zebrafish (Bielen and Houart, 2012). In the human retina, RAX2 is present in the outer and inner nuclear layers and serves as a PCE-1-binding protein, partnering with CRX and NRL to manage the expression of photoreceptor genes (Wang et al., 2004). Given the complex interplay among retinal cells and minor deviations may disrupt homeostasis, the deletion of *RAX2* could create cascading effects on retinal cell viability, thus affecting the progression of retinal development.

Our findings have significant translational relevance due to their potential for supporting retinal diseases treatments involving photoreceptor loss, such as retinitis pigmentosa (Klymenko et al., 2024) and AMD (Tan et al., 2023). RAX2 expression modulation may provide dual effects of both preventing photoreceptor degeneration and promoting their regeneration. Future studies should investigate the role of RAX2 in ocular development, develop therapies by expressing the human *RAX2* gene in Rax-deficient mice, and generate disease models using RO. Understanding RAX2’s interactions with key developmental genes like *PAX6* and *SOX2* is crucial for advancing gene therapy approaches for retinal disorders.

Our study acknowledges limitations in fully delineating the molecular interactions of RAX2. Future research using advanced genetic techniques and precise temporal analysis will be essential for elucidating the detailed mechanisms underlying this genetic pathway in retinal development. This study highlights the importance of further exploring the regulatory functions and interactions of RAX2 to improve our comprehension of retinal development and discover new therapeutic interventions for retinal disorders linked to these cells.

## Data Availability

The datasets presented in this study can be found in online repositories. The names of the repository/repositories and accession number(s) can be found in the article/Supplementary Material.
