# Supplementary material for: Molecular analysis of RAX2-regulated retinal development using human retinal organoids at a single-cell resolution
Source: Front Cell Dev Biol. 2025 Jun 5;13:1609826. doi: 10.3389/fcell.2025.1609826 (PMC12176875; doi:10.3389/fcell.2025.1609826)
Supplement: Supplementary file 1 [file DataSheet1.docx]

Supplementary Material

# Supplementary Material

**1.1 Multiplex immunofluorescence**

Retinal organoids (RO) at different time points (D36, D66, D96, D126, and D186), along with human fetal retina tissue, were fixed in 4% paraformaldehyde (PFA), then embedded in paraffin and sectioned into 5 μm slices. Dewaxed slices were deparaffinized twice in xylene (5 min each), and then hydrated with a different gradient alcohol. Multiplex immunofluorescence was conducted with the TSA-RM-275 IHC kit (Panovue, 10001100020). Primary antibodies: Rabbit anti-RAX2 (Invitrogen, PA5-144014), Rabbit anti-OTX2 (Abcam, ab183951), Rabbit anti-PAX6 (Abcam, ab195045) and Rabbit anti-SOX2(Abcam, ab92494). Images were captured with a Leica Stellaris 5 confocal microscope.

**1.2 RO differentiation from hESC**

The RO differentiation was performed followed the previously published protocol with minor modifications(Wang et al., 2021, Kuwahara et al., 2015). On day 0 (D0), single cells were seeded at a density of 12,000 cells per well in 100 μL of growth factor-free chemically defined medium (gfCDM) supplemented with 20 μmol/L Y-27632 (MCE, HY-10071) using low-adhesion V-bottom 96-well plates, and cultured at 37°C under 5% CO₂. The gfCDM contains 45% IMDM (Gibco, 12440053), 45% Ham’s F-12 (Gibco, 31765035), 1% GlutaMAX (Gibco, 35050061), 1% CD lipid concentrate (Gibco, 11905031), 450 μM 1-thioglycerol (Sigma, M6145), and 100 U/mL penicillin–streptomycin (Gibco,15140122). On D6, recombinant human BMP4 (Peprotech, AF-120-05ET) was added to a final concentration of 1.5 nM, followed by half-medium replacement every 3 days. From D24 onward, aggregates were transferred to low-adhesion 10-cm dishes (30 aggregates/dish) in NR-differentiation medium comprising DMEM/F12 (Gibco, 10565018), KSR (Gibco, 10828028), N2 supplement (Gibco, A1370701), 0.1 mM taurine (Sigma, T0625), and 0.5 μM retinoic acid (Sigma, R2625) under 40% O2/5% CO2 conditions. Under these conditions, RO continued to grow for several weeks.

**1.3 Immunofluorescence of hESC**

Cells were rinsed three times with DPBS, fixed in 4% paraformaldehyde (PFA) for 20 min at room temperature (RT), and permeabilized with 0.1% Triton X-100 for 15 min at RT. Subsequently, cells were blocked with 3% bovine serum albumin (Sigma, B2064) in DPBS for 60 min at RT. Primary antibodies were diluted in blocking buffer and incubated overnight at 4°C. Primary antibodies: Rabbit anti-NANOG (HUABIO, ET16110-2) and Rabbit anti-OCT4(Cell Signaling Technology, 2840). The cells were then incubated with secondary antibodies in blocking buffer for 60 min at RT. Secondary antibodies: Alexa Fluor 488 donkey anti-rabbit IgG (Invitrogen, A-21206). Nuclei were counterstained with DAPI (Invitrogen, S3693). Imaging was performed using a Leica Stellaris 5 confocal microscope equipped with 10× objective.

**1.4 Alkaline phosphatase** **staining**

Cells were cultured at low density for 5 days, rinsed with DPBS and fixed with 4% PFA for 2 min at RT. Staining was performed with an alkaline phosphatase kit (Sigma, 86R-1KT), adhering to the protocol provided by the manufacturer. Images were taken by an Olympus X41inverted microscope, using a 10×objective.

**1.5 CCK8 assay**

Cells were placed at a concentration of 5,000 per well in a 96-well plate and maintained in Essential 8 medium. We refreshed the medium at 24-hour intervals, specifically at 24h, 48h, 72h, 96h and 120 hours, by introducing new medium enriched with 10µl of CCK-8 solution (DOJINDO, CK04-01) into 100 µl of Essential 8 medium. After two hours of incubation, the absorbance of the plate was recorded at 450 nm and 600 nm using the Synergy H1 Microplate Reader (BioTek, Dallas, TX, USA).

**1.6 Genomic copy number analysis**

Whole-genome libraries prepared with the NEBNext® Ultra™ II kit (NEB, E7645L) were sequenced on a NovaSeq 6000 to produce 2 × 150 bp PE reads. Initial read pro-cessing, involving quality control and adapter removal, was conducted via fastp (v0.20.0). These reads were then mapped to the hg19 genome using BWA (v0.7.12), with the subsequent BAMs organized and deduplicated by SAMtools (v1.4) and Sambamba (v0.6.6). CNV analysis was performed with Control-FREEC (v11.4), excluding the Y chromosome.

**1.7 EB formation**

The hESC were dissociated into single cells and then plated at a concentration of 3,000 cells per well in an AggreWell™800 plate (Stemcell Technologies, 34811). These cells were cultured in EB formation medium (Stemcell Technologies, 07010), with the medium being refreshed every other day. The formation of EBs was recorded under the Zeiss Axio Observer5 microscope using a 5×objective on days 6 and 12, EBs were also harvested on day6 and day12 for RNA extraction to evaluate the differentiation into ectoderm, endoderm and mesoderm lineages.

**1.8 RNA extraction and quantitative reverse transcription PCR (RT-qPCR)**

Total RNA was extracted with TRIzol reagent (Invitrogen, 10296010), and reverse transcription was subsequently conducted with the PrimeScript II 1st Strand cDNA Syn-thesis Kit (TaKaRa, D6210A). RT-qPCR was conducted with TB Green Premix Ex Taq (TaKaRa, RR820Q) on Roche Light Cycler 480® System. ACTIN or GAPDH served as in-ternal controls to normalize the relative expression values. All the primers used were listed in Supplementary Table 1.

**1.9 Preparation and sequencing of single-cell cDNA library**

RO at D66 were collected, digested, and resuspended in DPBS, following the procedure previously described (Qiu et al., 2017). In this work, twenty-four RO were harvested from each of WT and *RAX2*^−/−^ group. Given the similar genomic features and stem cell properties of the two *RAX2*^−/−^ hESC strains, RO derived from these two *RAX2*^−/−^ hESC were pooled for scRNA-seq analysis.

**1.10 Single-cell RNA-sequencing data analysis**

For single cell data preprocess, the initial sequencing data quality was evaluated using FastQC software. After trimming, the fastq sequences were processed through the Cell Ranger (2.0.1) pipeline to create a single cell matrix for each sample, which were then merged using the Seurat R package (Butler et al., 2018).

For clustering analysis, feature selection involved identifying top genes based on dispersion across 15 gene expression distribution bins. The features were adjusted according to the percentage of mitochondrial genes and total number of unique molecular identifiers (UMI) in each cell. Principal Component Analysis (PCA) was conducted on scaled values of the 2,000 genes with the highest variability. An elbow plot was used to identify the optimal number of principal components. We scrutinized the gene loadings on the principal components and excised those that demonstrated substantial variance among cells. A network of nearest neighbors was generated by defining a fixed number of neighbors and using Euclidean distances. After adjusting for total cell UMI counts and mitochondrial gene percentages, we reduced dimensionality with UMAP using the refined principal components. We then conducted clustering using the Louvain method within the Seurat package (Butler et al., 2018). Using Seurat's Find Markers function, cell type markers were pinpointed, and unique gene markers for each cluster were analyzed. The Scanpy Python package was utilized to identify subpopulations (Wolf et al., 2018). We subset the data for the targeted population and then reclustered the cells using the UMAP technique.

For single cell trajectory analysis, using the SCORPIUS R package

(Wolf et al., 2018), we analyzed single-cell trajectories with a focused matrix of cell and gene expressions. Pearson’s correlation facilitated dimensionality reduction. Candidate gene markers (P value<0.05) were grouped into modules reflecting dynamic expression patterns, which were then visualized in a trajectory heatmap.

For gene ontology (GO) analysis, enrichments were performed by Metascape and Gene Ontology Resource databases. The online tool Enrichr was used to perform these analyses and visualization. Differentially expressed genes were distinguished by having an adjusted p-value less than 0.05 and a log2(fold-change) over 0.5.

The raw sequence data reported in this paper have been deposited in the Genome Sequence Archive(Chen et al., 2021) in National Genomics Data Center(Members and Partners, 2022), China National Center for Bioinformation / Beijing Institute of Genomics, Chinese Academy of Sciences (GSA-Human: HRA011550) that are publicly accessible at <https://ngdc.cncb.ac.cn/gsa-human>.

**1.11 Statistical analysis**

Statistical analysis was performed using the student's t-test. Data was presented as means ± SD. P-value above 0.05 was considered not statistically significant, whereas p-value below 0.05 was considered statistically significant.

# Supplementary Figures and Tables

##
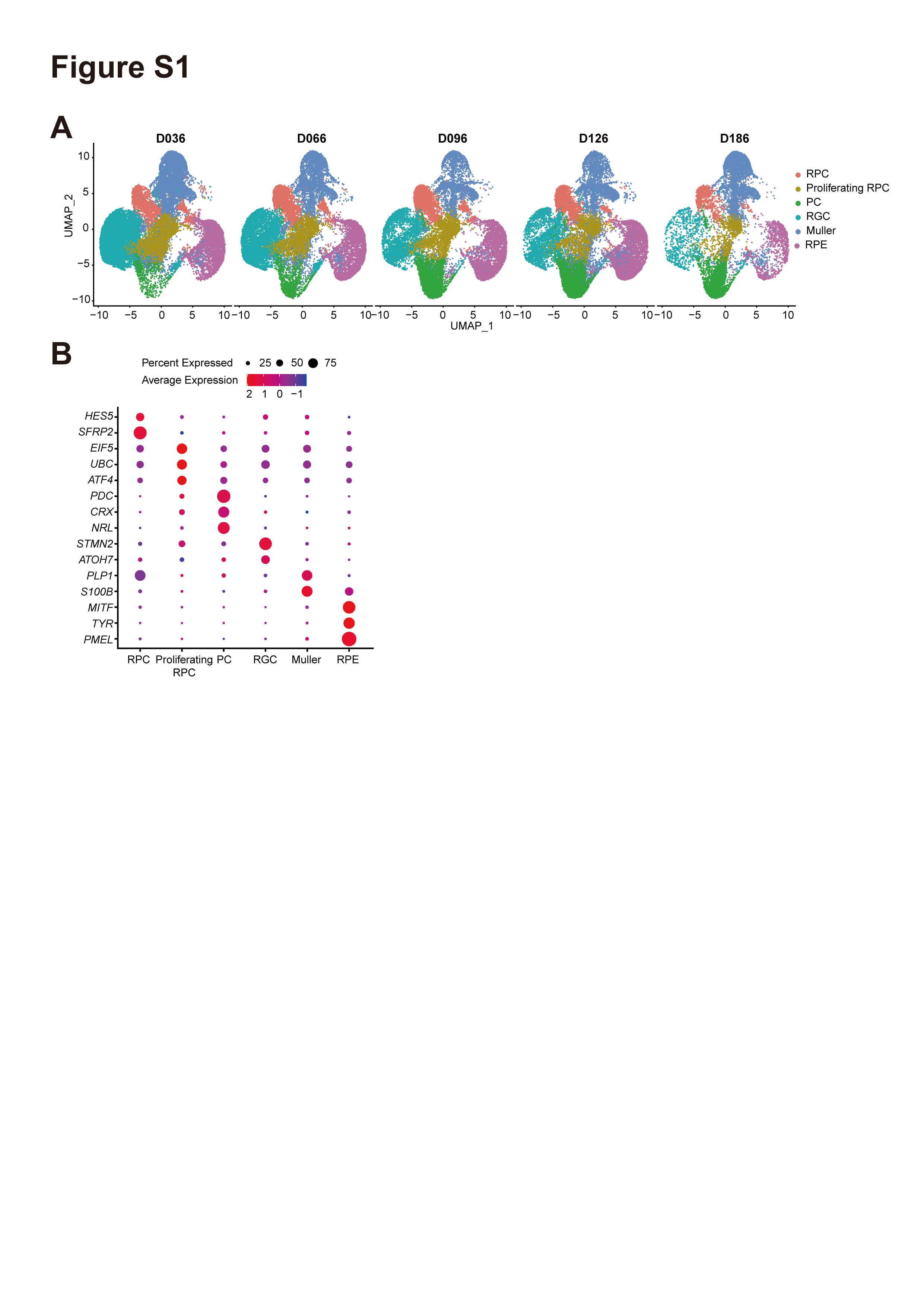
Supplementary Figures

**Supplementary Figure 1. Cells clusters in hESC-derived RO.** **(A)** UMAP plots of the single-cell RO dataset, labeled by clusters and different timepoints (D36, D66, D96, D126, D186). RPC, retinal progenitor cells; Proliferating-RPC; PC, photoreceptor precursor cells; RGC, retinal ganglion cells; MC, Müller cell; RPE, retinal pigment epithelial. **(B)** Dot plots exhibiting marker genes expression for each cell cluster. **(C)** Fluorescence intensity profiles of RAX2 and CRX in hESC-derived RO at different timepoints (D036, D096, D126). **(D)** Fluorescence intensity profiles of RAX2 and OTX2 in human retinal tissue from aborted fetuses, spanning gestational ages of 12 to 24 weeks.


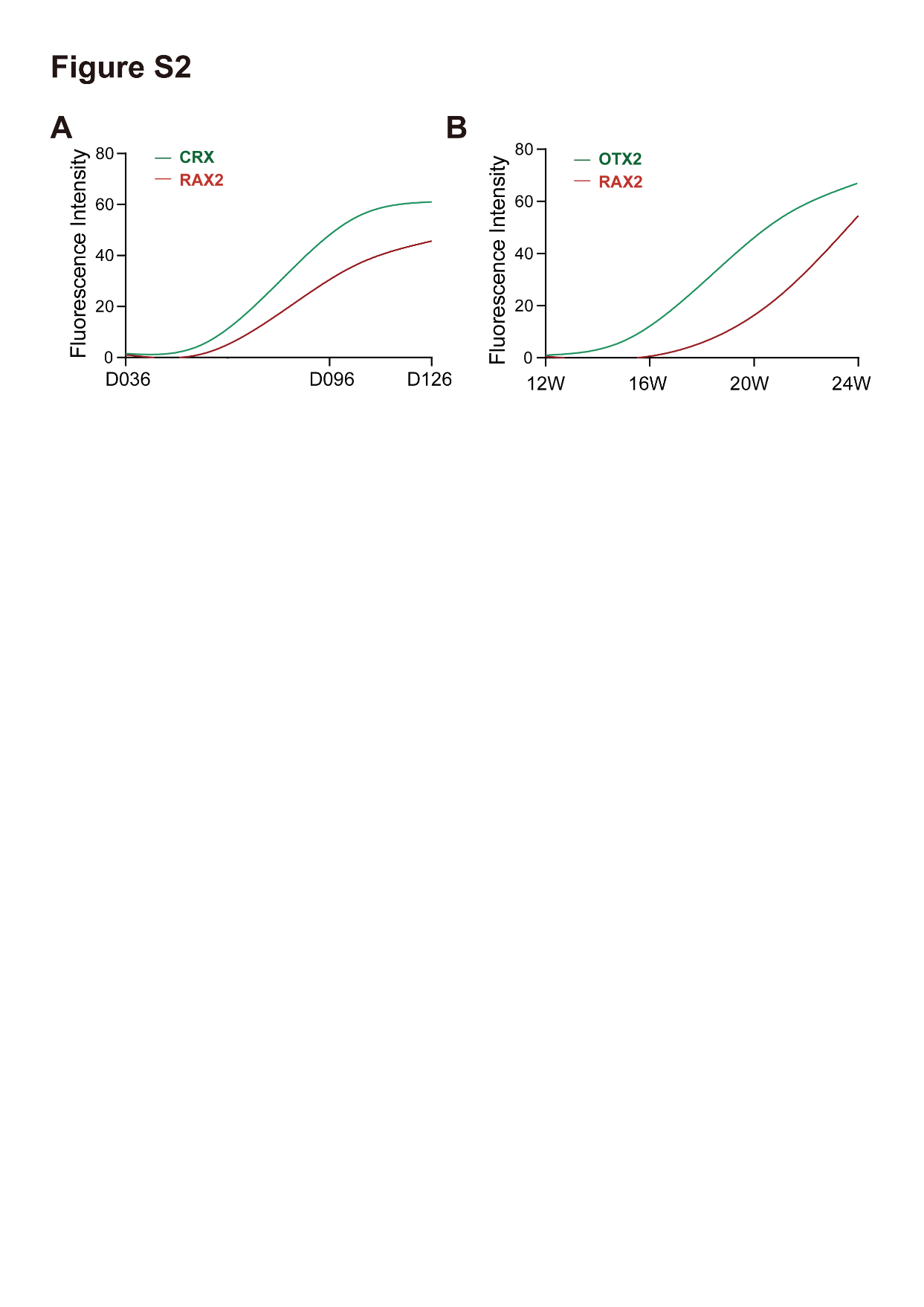


**Supplementary Figure 2.** **The IF signal intensities of RAX2 along with the other markers in RO and human fetal retinal samples at different time points.** **(A)** Fluorescence intensity profiles of RAX2 and CRX in hESC-derived RO at different timepoints (D036, D096, D126). **(B)** Fluorescence intensity profiles of RAX2 and OTX2 in human retinal tissue from aborted fetuses, spanning gestational ages of 12 to 24 weeks.

**
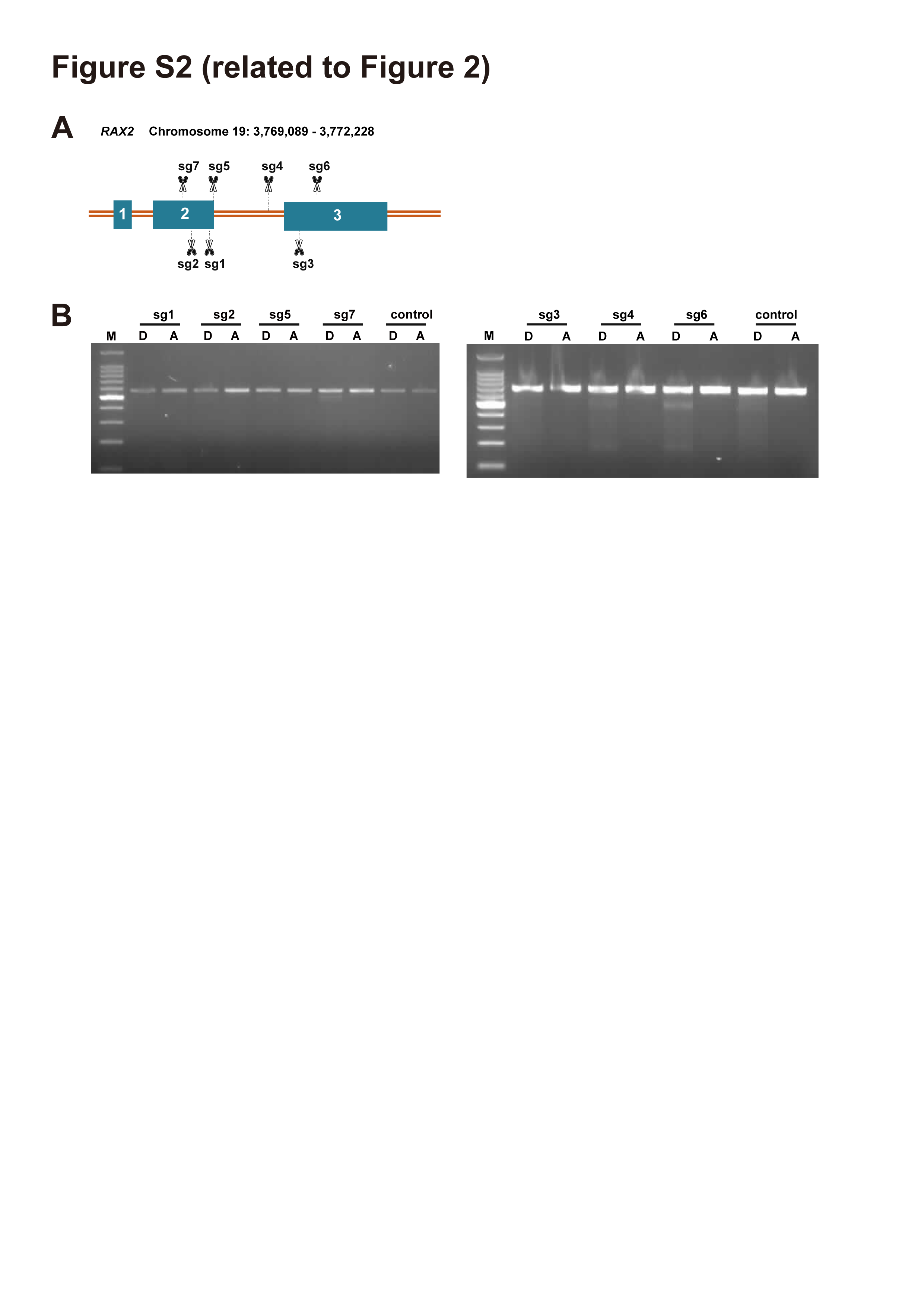
**

**Supplementary Figure 3.** **Surveyor assay for evaluating the effectiveness of multiple sgRNAs targeting the *RAX2* gene. (A)** Seven single guide RNAs (sgRNAs) targeting different sites up- or downstream of the region of *RAX2*. **(B)** Surveyor assay for Cas9-mediated cleavage up- and downstream of the *RAX2* locus in hESC. PCR products were denatured, annealed and digested with T7 endonuclease I (T7EN1). Lane M: Standard molecular size marker (100 bp); lane D: T7EN1 digest products; lane A: annealed products without T7EN1 digest.


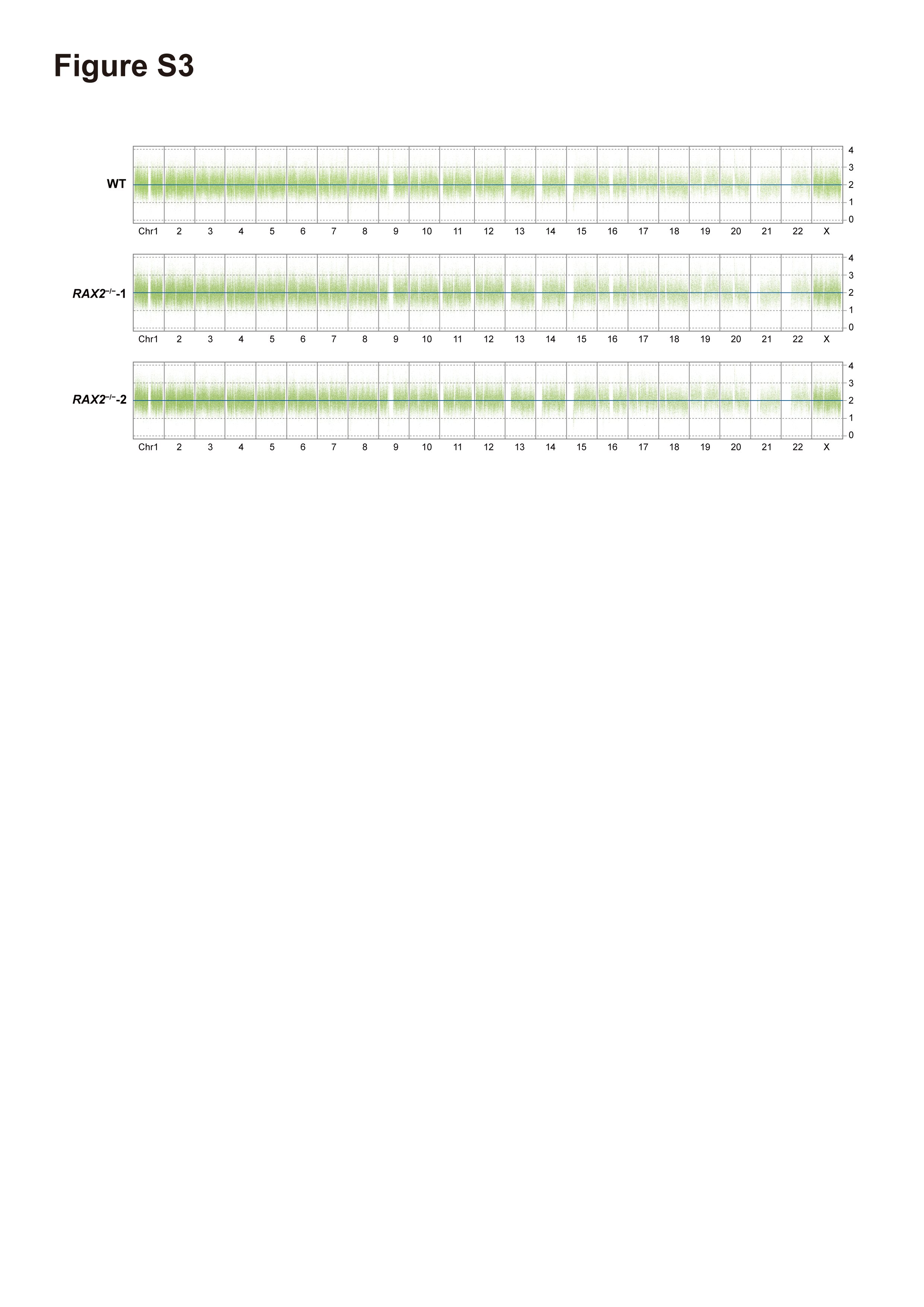


**Supplementary Figure 4.** **Genomic copy number variation (CNV) assay of *RAX2*^−/−^ and WT hESC clones.**


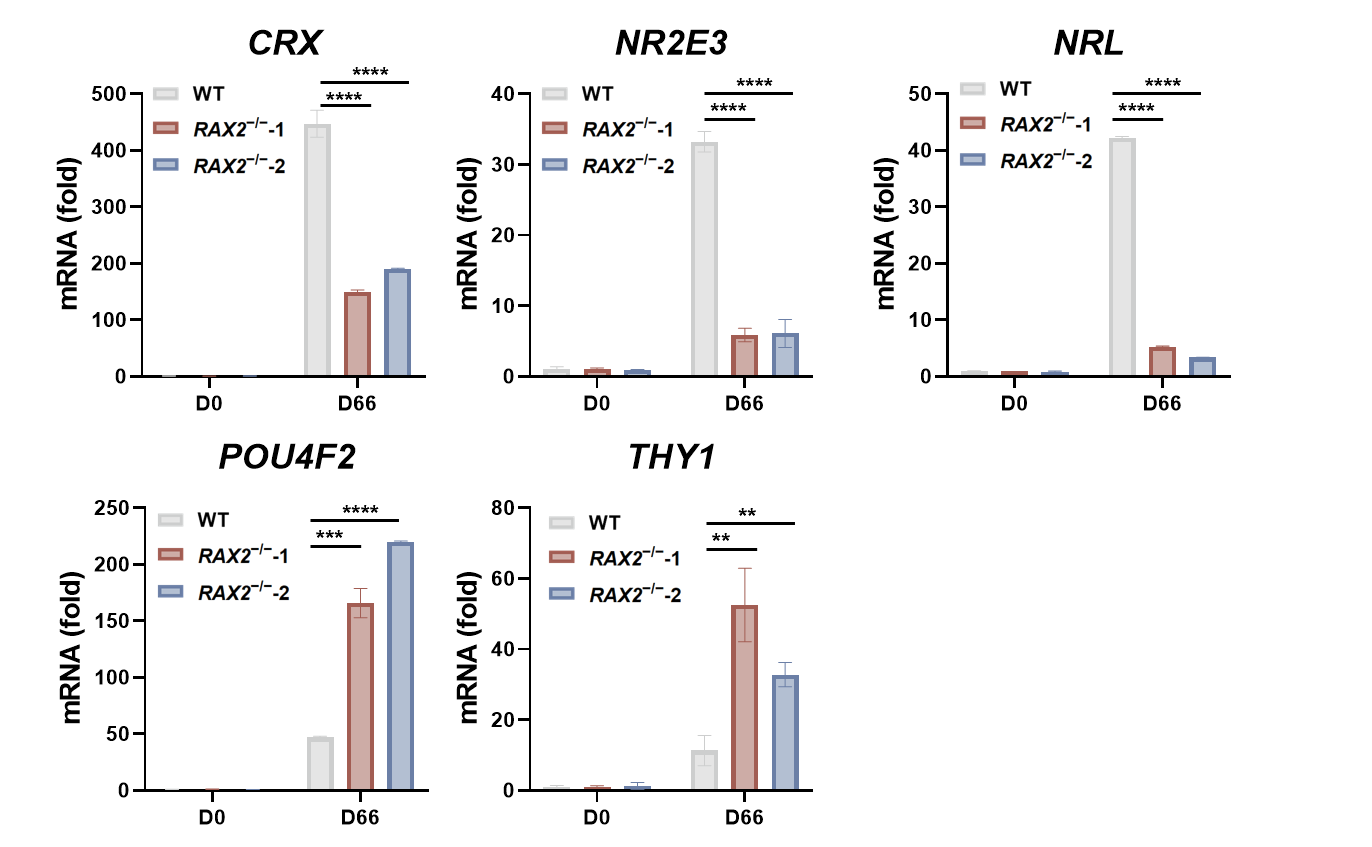
**Supplementary Figure 5. RT-qPCR analysis for RGC and PC markers expression in WT and *RAX2*^−/−^ hESC-derived RO (D66).**

**2.2 Supplementary Table 1.** DNA sequences of Oligonucleotides used in this study. Related to Experimental Procedures.

| Name | Sequence (5’ to 3’) | Purpose |
| --- | --- | --- |
| *RAX2*-sg1 | GTACACATCCGGGTAGTGAG | Gene knockout |
| *RAX2*-sg2 | GGGCCGGCAACCGAGGGTGG |  |
| *RAX2*-sg3 | GCTGGCACGGCCGGCGGTCCG |  |
| *RAX2*-sg4 | GAGGGGGTCCGAGGATTGGCG |  |
| *RAX2*-sg5 | GCCTACCTGAGGTGCGCGTGC |  |
| *RAX2*-sg6 | GCTTCGCAGATGGCTTCGCCC |  |
| *RAX2*-sg7 | GTGTTCCTGAGCCCGGGCGAG |  |
| *POU5F1*-Fw | CGACCATCTGCCGCTTTGAG | RT-qPCR |
| *POU5F1*-Rv | CCCCCTGTCCCCCATTCCTA |  |
| *NANOG*-Fw | CAGTCCTGGAGCAACCACTC |  |
| *NANOG*-Rv | ACATTAAGGCCTTCCCCAGC |  |
| *PAX6*-Fw | AACGATAACATACCAAGCGTGT |  |
| *PAX6*-Rv | GGTCTGCCCGTTCAACATC |  |
| *MAP2*-Fw | AGGAAGGGAAGAGCATGTAA |  |
| *MAP2*-Rv | ACACCACTTCCTCAACCACAG |  |
| *RAX*-Fw | GCAAGGTCAACCTACCAGAGGT |  |
| *RAX*-Rv | GCAGCTTCATGGAGGACACTTC |  |
| *SIX6*-Fw | AGAATGAGTCGGTGCTACGC |  |
| *SIX6*-Rv | GCCTCCTGGTAGTTGTGCTTC |  |
| *T*-Fw | TATGAGCCTCGAATCCACATAGT |  |
| *T*-Rv | CCTCGTTCTGATAAGCAGTCAC |  |
| *GATA4*-Fw | GTGTCCCAGACGTTCTCAGTC |  |
| *GATA4*-Rv | GGGAGACGCATAGCCTTGT |  |
| *HAND1*-Fw | CCATGCTCCACGAACCCTTC |  |
| *HAND1*-Rv | CCTGGCGTCAGGACCATAG |  |
| *SOX17*-Fw | CTGCCACTTGAACAGTTTGG |  |
| *SOX17*-Rv | GAGGAAGCTGTTTTGGGACA |  |
| *RAX2*-Fw | CTCCCCAGATCACTCCCAGA |  |
| *RAX2*-Rv | TCTTGTGCTTACTGCCGTGT |  |
| *SOX2*-Fw | GGGAAATGGGAGGGGTGCAAAAGAGG |  |
| *SOX2*-Rv | TTGCGTGAGTGTGGATGGGATTGGTG |  |
| *β-ACTIN*-Fw | CCACGAAACTACCTTCAACTCC |  |
| *β-ACTIN*-Rv | GTGATCTCCTTCTGCATCCTGT |  |
| *GAPDH*-Fw | TGATGACATCAAGAAGGTGGTGAAG |  |
| *GAPDH*-Rv | TCCTTGGAGGCCATGTGGGCCAT |  |

**3** **Supplementary References**

BUTLER, A., HOFFMAN, P., SMIBERT, P., PAPALEXI, E. & SATIJA, R. 2018. Integrating single-cell transcriptomic data across different conditions, technologies, and species. *Nat Biotechnol,* 36**,** 411-420.

CHEN, T., CHEN, X., ZHANG, S., ZHU, J., TANG, B., WANG, A., DONG, L., ZHANG, Z., YU, C., SUN, Y., CHI, L., CHEN, H., ZHAI, S., SUN, Y., LAN, L., ZHANG, X., XIAO, J., BAO, Y., WANG, Y., ZHANG, Z. & ZHAO, W. 2021. The Genome Sequence Archive Family: Toward Explosive Data Growth and Diverse Data Types. *Genomics Proteomics Bioinformatics,* 19**,** 578-583.

KUWAHARA, A., OZONE, C., NAKANO, T., SAITO, K., EIRAKU, M. & SASAI, Y. 2015. Generation of a ciliary margin-like stem cell niche from self-organizing human retinal tissue. *Nat Commun,* 6**,** 6286.

MEMBERS, C.-N. & PARTNERS 2022. Database Resources of the National Genomics Data Center, China National Center for Bioinformation in 2022. *Nucleic Acids Res,* 50**,** D27-D38.

QIU, X., HILL, A., PACKER, J., LIN, D., MA, Y. A. & TRAPNELL, C. 2017. Single-cell mRNA quantification and differential analysis with Census. *Nat Methods,* 14**,** 309-315.

WANG, S., POLI, S., LIANG, X. & PENG, G. H. 2021. Longitudinal single-cell RNA-seq of hESCs-derived retinal organoids. *Sci China Life Sci,* 64**,** 1661-1676.

WOLF, F. A., ANGERER, P. & THEIS, F. J. 2018. SCANPY: large-scale single-cell gene expression data analysis. *Genome Biol,* 19**,** 15.
